# Perspectives on the importance of parents’ health, health-promoting behaviour, and psychosocial and lifestyle factors during pregnancy on child health outcomes across the life course: a cross-sectional study among parents and professionals

**DOI:** 10.1093/pubmed/fdaf133

**Published:** 2025-10-22

**Authors:** Sushma C Munshi, Loes C M Bertens, Anne Marie Weggelaar-Jansen, Hiske E Ernst-Smelt, Mijke P Lambregtse-van den Berg, Hanneke W Harmsen van der Vliet-Torij, Eric A P Steegers, Hilmar H Bijma

**Affiliations:** Department Obstetrics and Gynaecology, Division Obstetrics and Fetal Medicine, Erasmus MC—Sophia Children’s Hospital, University Medical Centre Rotterdam, Dr. Molewaterplein 40, 3015 GD Rotterdam, South Holland, The Netherlands; Department Obstetrics and Gynaecology, Division Obstetrics and Fetal Medicine, Erasmus MC—Sophia Children’s Hospital, University Medical Centre Rotterdam, Dr. Molewaterplein 40, 3015 GD Rotterdam, South Holland, The Netherlands; Tranzo, Tilburg University, Professor Cobbenhagenlaan 125, 5000 LE Tilburg, North Brabant, The Netherlands; Department Obstetrics and Gynaecology, Division Obstetrics and Fetal Medicine, Erasmus MC—Sophia Children’s Hospital, University Medical Centre Rotterdam, Dr. Molewaterplein 40, 3015 GD Rotterdam, South Holland, The Netherlands; Department of Psychiatry and Child and Adolescent Psychiatry, Erasmus MC—University Medical Centre Rotterdam, Dr. Molewaterplein 40, 3015 GD Rotterdam, South Holland, The Netherlands; Research Centre Innovations in Care, Rotterdam University of Applied Sciences, Rochussenstraat 198, 3015 EK Rotterdam, South Holland, The Netherlands; Department Obstetrics and Gynaecology, Division Obstetrics and Fetal Medicine, Erasmus MC—Sophia Children’s Hospital, University Medical Centre Rotterdam, Dr. Molewaterplein 40, 3015 GD Rotterdam, South Holland, The Netherlands; Department Obstetrics and Gynaecology, Division Obstetrics and Fetal Medicine, Erasmus MC—Sophia Children’s Hospital, University Medical Centre Rotterdam, Dr. Molewaterplein 40, 3015 GD Rotterdam, South Holland, The Netherlands; Department of Care Ethics, University of Humanistic Studies, Kromme Nieuwegracht 29, 3512 HD Utrecht, Utrecht, The Netherlands

**Keywords:** parental health, pregnancy, child health, health behaviour, psychosocial factors, lifestyle, health promotion, public health, early life course

## Abstract

**Background:**

Adverse circumstances during pregnancy are associated with impaired health for children not only during pregnancy and childhood, but also in adulthood. This study evaluates the perspectives of parents and professionals regarding the importance of parents’ health, parents’ health-promoting behaviour, and psychosocial and lifestyle factors of parents during pregnancy on a child’s long-term health outcomes.

**Methods:**

A cross-sectional survey study was conducted among parents with a child up to two years (n = 1854) and professionals (n = 322) in a large city in the Netherlands.

**Results:**

Most parents and professionals agree that maternal health during pregnancy is important for a child’s health during pregnancy (98%, 99%, respectively), childhood (94%, 97%, respectively), and adulthood (84%, 89%, respectively). Additionally, almost all parents and professionals agree that maternal health-promoting behaviour during preconception (90%, 96%, respectively), pregnancy (97%, 98%, respectively), and childhood (97%, 99%, respectively) is important for a child’s health.

**Conclusion:**

Most parents and professionals recognize the importance of parents’ health and well-being, parents’ health-promoting behaviour and psychosocial and lifestyle factors of parents during pregnancy for a child’s health throughout the life course. To optimize public health, there is a need for effective knowledge translation to bridge between recognition and health-promoting behaviour.

## Introduction

Adverse circumstances during the early life course (ELC)—ranging from 100 days before conception until the first 1000 days thereafter (two years after birth)—have been associated with non-communicable diseases (NCDs) at adult age.^1^^,^^2^ The ELC shapes adult health through a complex interaction of genetic, psychosocial, and lifestyle factors.^3^ Adverse circumstances during the ELC significantly increase the risk of NCDs later in life.^4^ For instance, maternal poor nutrition, poverty, neighbourhood deprivation, substance use, and/or a history of adverse early childhood experiences during pregnancy are associated with increased risk of NCDs at adult age, such as obesity, type 2 diabetes, cardiovascular and respiratory disease, cancer, mental illness, metabolic syndrome, vulnerability to chronic health conditions, and negative impact on socioeconomic life opportunities.^5–14^ Consequently, the unfavourable effects of adverse circumstances can be passed on from generation to generation, resulting in intergenerational cycles of increasing health inequality.^15^^,^^16^

The importance of improving adverse circumstances during the ELC for public health is increasingly recognized in public health policy-making, resulting in many countries adopting programs aiming at improving these circumstances.^17^^,^^18^ However, insights into the perspectives of parents with a child up to two years (hereafter: parents) and maternal and child healthcare professionals engaged in ELC care (hereafter: professionals) on the long-term impact of circumstances during the ELC for a child’s adult health remain limited. To date, limited existing studies, varying in approach and target groups, examined perspectives on the importance of parents’ general health or specific psychosocial, lifestyle, or biological factors of parents during the ELC on a child’s health throughout life, resulting in a fragmented understanding.^19^ Insights into these perspectives of parent and professionals regarding the importance of early life circumstances in shaping not only a child’s immediate health, but also their long-term adult health are critical for the development of public health policies aimed at improving overall health, reducing NCDs, and addressing health inequalities. These insights are essential for effective knowledge translation, ultimately improving public health of both current and future generations.^20–22^

Therefore, this study aims to (i) evaluate the perspectives of parents and professionals regarding the importance of parents’ general health and well-being, parents’ health-promoting behaviour, and psychosocial and lifestyle factors of parents during the ELC—i.e. preconception, pregnancy and childhood—on a child’s health and well-being during multiple life course stages—i.e. pregnancy, childhood, and adulthood; (ii) identify characteristics associated with these perspectives; and (iii) evaluate differences in perspectives between parents and professionals.

## Methods

### Study design and study participants

A cross-sectional city-based population survey study was conducted in the wider city of Rotterdam, the Netherlands by a research collaborative^23^ involving public, medical, and social care professionals from seven organisations who jointly provide the majority of public health, medical, and social care to (future) children from preconception until the age of two years and their parents in Rotterdam. All (registered) parents or primary caregivers of children up to two years in Rotterdam were invited to participate. A survey link was sent via email by the public health care organization providing preventive youth healthcare, which serves all children until the age of 18 years. In the email, parents were addressed personally (e.g. ‘Parents of [name of child]’) and invited to complete the survey. Professionals were invited to participate via email which was distributed by organisations of the research collaborative.

### Data collection

**Figure 1:**
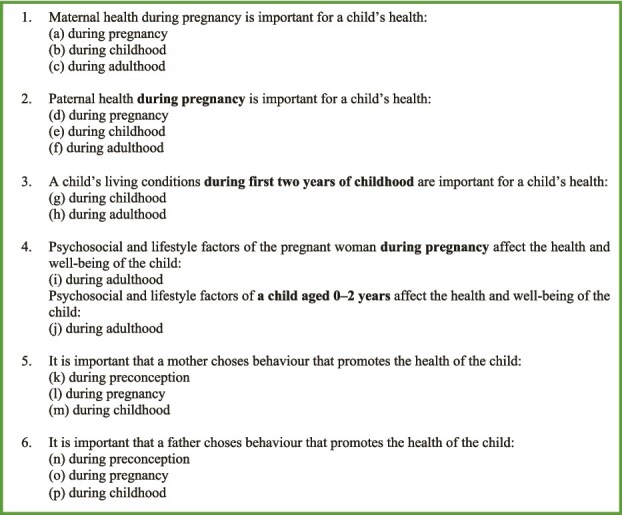
Statement sets 1–6 regarding parents’ general health and well-being, parent’s health-promoting behaviour, and psychosocial and lifestyle factors of parents during ELC.

Data were collected using a survey that was developed based on previous studies,^24^^,^^25^ expert opinions, and lived experiences of parents with and without psychosocial adversity. The survey items were presented in statement sets per theme or the ELC time period (see Fig. 1), with participants only being able to respond to a new statement set if the previous had been responded to. Participants did have an option to move back to a previous statement set and had the option to ‘skip this question’ for each statement. Two parents and 16 professionals with and without maternal and child healthcare backgrounds evaluated the survey’s content, format, length, and understandability in three iterative cycles resulting in adjustments. Subsequently, the survey was further refined in a pilot study involving 75 participants, including medical students and citizens, to ensure understandability.

The survey evaluated participants’ perspectives by asking to what extent participants agreed with statements on the importance of parents’ general health and well-being (hereafter: maternal or paternal health), of multiple psychosocial and lifestyle factors of parents, and parents’ health promoting behaviour during the ELC on a child’s health and well-being (hereafter: child's health) throughout the life course. Statement agreement was evaluated using a 5-point Likert scale, with the response categories: strongly agree, somewhat agree, neither agree nor disagree, somewhat disagree or strongly disagree. The survey comprised of four sections related to the importance of (see Fig. 1): (i) parents’ general health during pregnancy for a child’s health during pregnancy, childhood, and adulthood; (ii) child’s living conditions during the first two years of childhood for a child’s health during childhood and adulthood; (iii) psychosocial and lifestyle factors of parents during pregnancy and childhood for a child’s health during adulthood; and (iv) parents’ health-promoting behaviour during preconception, pregnancy, and childhood for a child’s general health (see Appendix A for the translated survey). Additionally, participants’ socio-demographic characteristics were collected.

### Determinants

The response categories ‘strongly agree’ and ‘somewhat agree’ of the Likert scale were combined for analysis into a group ‘agree’. The determinants—parents’ general health, psychosocial, and lifestyle factors of parents and parents’ health-promoting behaviour—were evaluated through statements addressing life course stages preconception, pregnancy and childhood. (Future) child health outcomes were grouped by the life course stages pregnancy, childhood, and adulthood. Each question included the option to be skipped, and any question skipped by participants was treated as missing data. For parents, gender was categorized as male, female, or other. Country of birth was dichotomized as born in the Netherlands or elsewhere. (Receiving professionals support for) psychosocial adversity was defined as experiencing difficulties regarding finances, housing, relationships, mental health, substance use, social support, language comprehension, as well as having experienced abuse or neglect, or severe and persistent stress. Psychosocial adversity and receiving professional support for these adversities were evaluated through two non-mandatory multiple-response questions due to their sensitive nature, each including an answer option ‘none’, which resulted in 563 (30%) and 540 (29%) missing responses, respectively. For professionals, years of professional experience was categorized as ‘0–5 years’, ‘5–10 years’, ‘10–20 years’, ‘20–30 years’, or ‘more than 30 years’. Weekly working hours was categorized as ‘less than 16 hours’, ‘16–24 hours’, ‘25–32 hours’, ‘33–40 hours’, or ‘more than 40 hours’.

### Statistical analysis

Descriptive results are presented as frequency and percentages for categorical variables, and means and standard deviations or median and interquartile ranges for continuous variables as appropriate. Differences in proportions of agreement between statements within a statement set regarding child health outcomes across the life course were tested with paired simple proportions t-test. To achieve this, we included only those parents and professionals who provided responses to all statements per statement set concerning child health outcomes across the life course (e.g. for statement set 1 that is a-c, see Fig. 1). Differences in agreement between parents and professionals were tested with chi-squared tests. Binary logistic regressions models were used to estimate the odds ratios (OR) and 95% confidence intervals (95% CI) for the association between parents’ and professionals’ characteristics and agreement. Specifically, we conducted a multivariate logistic regression using a backwards stepwise method based on the Wald statistic, applied in three separate blocks. For parents, we examined gender, age, relationship status, current concomitant wish to conceive or pregnancy, and (received additional support) for psychosocial adversity. For professionals, we examined age, years of professional experience, working hours per week, and professional area. Sensitivity analyses were conducted combining missing responses with either the group that did agree (i.e. all responses that ‘strongly agree’ or ‘agree’) or the group that did not agree, to assess the potential impact of missing responses on the overall findings (see Supplementary Tables 12–17). The sensitivity analyses showed that the overall conclusions were not significantly impacted by the missing data. All analyses were conducted using SPSS, version 28.1.0.1 (42), with tests of significance at the level of 0.05.

## Results

### Response rate

All 18 774 (registered) parents with a child up to two years were invited to participate by e-mail, with 2% of the e-mails being undeliverable. Of the remaining, 42.8% clicked the survey link, but did not submit any answers. The response rate, based on the unique clicks, was 10.1% resulting in 1854 parents being included in the analysis.

A total of 822 professionals involved in ELC-care were invited to participate by e-mail. The response rate was 39.2% resulting in 322 professionals being included in the analysis.

### Socio-demographic characteristics

Parents’ mean age was 33.6 (SD 4.6) years, with 91% being female and 92% being married or living together. Most parents held an undergraduate (36%) or university degree (34%). Of the 70% of parents who responded to the question regarding psychosocial adversity, 349 (27%) psychosocial adversities were reported. Among the 71% of parents who answered the question about receiving professional support for psychosocial adversity, 10% indicated they had received such support for (multiple) psychosocial adversities. Remarkably, of the parents who experienced psychosocial adversity, 17% (n = 214) reported to receive no professional support (see Table 1).

Professionals’ mean age was 39.8 (SD 11.5) years, with 60% having over 5 years of professional experience and 78% working more than 25 hours per week, representing sectors such as obstetric care, midwifery care, public health, social care, health care, and care for intellectual disability. The majority of professionals worked during multiple (42%) or all stages of child development (23%) (see Table 2).

**Table 1:** Socio-demographic characteristics of parents and the general population of Rotterdam.

Parents (n, %)	1854	(100)	General population of Rotterdam (n, %)		
Age (in years; mean, SD)^a^	33.6	(4.6)	Age (n, % in age group 30–39 years)^k^	108 283	(16.1)
Gender (n, %)^b^			Gender (n, %, in age group 30–40 years)^l^		
Female	1680	(90.6)	Female	52 998	(15.6)
Male	171	(9.2)	Male	55 129	(16.5)
Other	1	(0.1)			
Education (n, %)^c^			Education (n, %)		
Post-secondary degree	442	(24.1)	Primary education, pre-vocational secondary education, secondary vocational education level 1^m^	155 910	(30.0)
Undergraduate degree	663	(36.2)	Senior general secondary education, pre-university education, secondary vocational education level 2-4^m^	193 740	(37.3)
University degree	617	(33.7)	Undergraduate degree and university degree^m^	169 940	(32.7)
Abroad	33	(1.8)			
Other	76	(4.2)			
Education partner (n, %)^d^					
Post-secondary degree	508	(29.2)			
Undergraduate degree	524	(31.0)			
University degree	528	(31.2)			
Abroad	49	(2.8)			
Other	130	(7.5)			
Relationship (n, %)^e^			Household composition (n, %)^n^		
Married/living together	1703	(92.3)	Two-person households	132 495	(19.7)
Single	76	(4.2)	One-person households	332 247	(49.4)
Together, but living apart	64	(3.5)			
Country of birth (n, %)^f^			Country of birth (n, %)^o^		
Netherlands	1613	(87.3)	Netherlands	301 515	(46.0)
Abroad	234	(12.6)	Abroad	353 953	(54.0)
Current intention to conceive or pregnancy (n, %)			Number of births per year (n, %)^p^	6836	(1.0)
Intention to conceive^g^	111	(6.0)			
Pregnancy^h^	63	(3.4)			
Psychosocial adversity (n, %)^j^	351	(27.1)			
Receiving professional support for psychosocial adversity (n, %)^j^	128	(9.7)			

a 12 missing values

b 2 missing values

c 23 missing values

d 115 missing values

e 11 missing values

f 7 missing values

g 27 missing values

h 112 missing values

i 563 missing values

j 540 missing values

k Demographic data based on the 2025 census data derived from a total population of 672 565 (source: Onderzoek010—Bevolking—Rotterdam)

l Demographic data based on the 2025 census data derived from a total population of 672 960 (source: Inwoners per gemeente | CBS)

m Demographic data based on 2023–2024 census data age < 27 years (source: Onderzoek010—Onderwijs—Rotterdam)

n Demographic data based on the 2025 census data derived from a total population of 519 590 (source: StatLine—Bevolking 15 tot 75 jaar; opleidingsniveau, wijken en buurten, 2023)

o Demographic data based on the 2022 census data derived from a total population of 655 468 (source: Dashboard—Bevolking—Rotterdam)

p Demographic data based on the 2024 census data derived from a total population of 670 425 (source: Onderzoek010—Bevolking—Rotterdam)

### Parents’ general health

The majority of parents and professionals agree that maternal health during pregnancy is important for a child’s health during pregnancy (98%, 99%, respectively), during childhood (94%, 97%, respectively), and during adulthood (84%, 89%, respectively). Both parents and professionals more often agree that maternal health during pregnancy is important for a child’s health during pregnancy than during childhood (*P* < .001, *P* = .008, respectively) or adulthood (*P* < .001, *P* < .0001, respectively) (see Tables 3 and 4).

**Table 2:** Socio-demographic characteristics of professionals.

Professionals	322	
Age (in years; mean, SD)^a^	39.8	(11.5)
Care domain (n, %)^b^		
Obstetric care	79	(35.7)
Special needs care	54	(24.4)
Preventive youth healthcare	51	(23.1)
Social care	32	(14.5)
Other	5	(2.3)
Years of professional experience (n, %)^c^		
0–5 years	99	(39.8)
5–10 years	54	(21.7)
10–20 years	61	(24.5)
20–30 years	18	(7.2)
Longer than 30 years	17	(6.8)
Period of care provision (n, %)^d^		
Preconception	8	(3.7)
Pregnancy and delivery	16	(7.4)
Pregnancy and the first two weeks of a child	13	(6.0)
First 2 years of a child	39	(18.1)
Multiple stages	90	(41.9)
All stages	49	(22.8)
Weekly working hours (n, %)^e^		
Less than 16 hours	2	(0.8)
16–24 hours	53	(21.3)
25–32 hours	94	(37.8)
33–40 hours	72	(28.9)
More than 40 hours	27	(10.8)

a 96 missing values

b 101 missing values

c 73 missing values

d 107 missing values

e 73 missing values

**Table 3:** Differences in proportion of parents’ agreement regarding the importance of parents’ general health during pregnancy and a child’s living conditions during first two years for a child’s health outcomes during pregnancy, childhood and adulthood.

	Pregnancy	Childhood	Sig.	Pregnancy	Adulthood	Sig.	Childhood	Adulthood	Sig.
Maternal health and well-being during pregnancy are important for a child’s health and well-being during^a^:	1631 (97.5%)	1564 (93.5%)	*P* < .001	1631 (97.5%)	1399 (83.6%)	*P* < .001	1564 (93.5%)	1399 (83.6%)	*P* < .001
Paternal health and well-being during pregnancy are important for a child’s health and well-being during^b^:	1257 (76.4%)	1274 (77.4%)	*P* = .290	1257 (76.4%)	1121 (68.1%)	*P* < .001	1274 (77.4%)	1121 (68.1%)	*P* < .001
A child’s living conditions during 0–2 years are important for a child’s health and well-being during^c^:							1612 (97.3%)	1572 (94.9%)	*P* < .001

a 181 missing values

b 209 missing values

c 198 missing values

^*^All values are percentages of parents stating strongly agree and somewhat agree (compared with neither agree nor disagree, somewhat disagree or strongly disagree)

The majority of parents and professionals agree that paternal health during pregnancy is important for a child’s health during pregnancy (77%, 88%, respectively), during childhood (77%, 89%, respectively), and during adulthood (68%, 81%, respectively). Both parents and professionals more often agree that paternal health during pregnancy is important for a child’s health during pregnancy than during adulthood (*P* < .001, *P* < .001, respectively) (see Tables 3 and 4).

### Psychosocial and lifestyle factors

The majority of parents and professionals agree that smoking (97%, 99%, respectively), severe and persistent stress (95%, 98%, respectively), living in poverty (79%, 90%, respectively) and living in an underprivileged neighbourhood (67%, 81%, respectively) during pregnancy influence a child’s health in adulthood (see Tables 5 and 6).

**Table 4:** Differences in proportion of professionals’ agreement regarding the importance of parents’ general health during pregnancy and a child’s living conditions during first two years for a child’s health outcomes during pregnancy, childhood and adulthood.

	Pregnancy	Childhood	Sig.	Pregnancy	Adulthood	Sig.	Childhood	Adulthood	Sig.
Maternal health and well-being during pregnancy are important for a child’s health and well-being during^a^:	295 (99.3%)	288 (97.0%)	*P* = .008	295 (99.3%)	264 (88.9%)	*P* < .001	288 (97.0%)	264 (88.9%)	*P* < .001
Paternal health and well-being during pregnancy are important for a child’s health and well-being during^b^:	262 (88.2%)	263 (88.6%)	*P* = .845	262 (88.2%)	240 (80.8%)	*P* < .001	263 (88.6%)	240 (80.8%)	*P* < .001
A child’s living conditions during 0–2 years are important for a child’s health and well-being during^c^:							293 (99.3%)	289 (98.0%)	*P* = .063

a 25 missing values

b 25 missing values

c 27 missing values

^*^All values are percentages of professionals stating strongly agree and somewhat agree (compared with neither agree nor disagree, somewhat disagree or strongly disagree)

**Table 5:** Differences in proportion of parents’ agreement regarding the influence of psychosocial and lifestyle factors of parents during pregnancy and during childhood for a child’s health outcomes during adulthood.

The influence of psychosocial and lifestyle factors on a child’s health and well-being in adulthood during:	Preconception	Pregnancy	Sig.	Pregnancy	Childhood	Sig.
Smoking^a^				1333 (96.5%)	1332 (96.5%)	*p* = 0.892
Severe and persistent stress^b^				1311 (94.7%)	1341 (96.9%)	*p* < 0.001
Living in poverty^c^				1082 (78.5%)	1086 (78.8%)	*p* = 0.768
Living in an unprivileged neighbourhood^d^				925 (67.2%)	893 (64.9%)	*p* = 0.023
(Maternal) nutrition^e^	1109 (77.5%)	1358 (94.9%)	*p* < 0.001			

^*^All values are percentages of parents stating strongly agree and somewhat agree (compared with neither agree nor disagree, somewhat disagree, or strongly disagree).

a 473 missing values

b 470 missing values

c 475 missing values

d 478 missing values

e 423 missing values

**Table 6:** Differences in proportion of professionals’ agreement regarding the influence of psychosocial and lifestyle factors of parents during pregnancy and during childhood for a child’s health outcomes during adulthood.

The influence of psychosocial and lifestyle factors on a child’s health and well-being in adulthood during:	Preconception	Pregnancy	Sig.	Pregnancy	Childhood	Sig.
Smoking^a^				275 (98.6%)	271 (97.1%)	*p* = 0.180
Severe and persistent stress^b^				273 (97.8%)	273 (97.8%)	*p* = 1.000
Living in poverty^c^				250 (89.6%)	249 (89.2%)	*p* = 0.804
Living in an unprivileged neighbourhood^d^				226 (81.0%)	224 (80.3%)	*p* = 0.678
(Maternal) nutrition^e^	247 (88.2%)	265 (94.6%)	*p* < 0.001			

a 43 missing values

b 43 missing values

c 43 missing values

d 43 missing values

e 42 missing values

^*^All values are percentages of maternal and child healthcare professionals stating strongly agree and somewhat agree (compared with neither agree nor disagree, somewhat disagree, or strongly disagree).

The majority of parents and professionals agree that (maternal) nutrition during preconception (78%, 88%, respectively) and during pregnancy (95%, 95%, respectively) influence a child’s health in adulthood. Both parents and professionals more often agree that (maternal) nutrition influences a child’s adult health during pregnancy than during preconception (*P* < .001, *P =* .001, respectively) (see Tables 5 and 6).

**Table 7:** Differences in proportion of parents’ agreement regarding the importance of parents’ health-promoting behaviour during preconception, pregnancy and childhood for a child’s (future) health and development.

	Preconception	Pregnancy	Sig.	Preconception	Childhood	Sig.	Pregnancy	Childhood	Sig.
It is important that a mother choses behaviour that promotes a (future) child’s health and development during^a^:	1385 (89.3%)	1499 (96.6%)	*p* < 0.001	1385 (89.3%)	1504 (97.0%)	*p* < 0.001	1499 (96.6%)	1504 (97.0%)	*p* = 0,286
It is important that a father choses behaviour that promotes a (future) child’s health and development during^b^:	1317 (85.3%)	1361 (88.1%)	*p* = 0.001	1317 (85.3%)	1479 (95.8%)	*p* < 0.001	1361 (88.1%)	1479 (95.8%)	*p* < 0.001

a 303 missing values

b 310 missing values

^*^All values are percentages of parents stating strongly agree and somewhat agree (compared with neither agree nor disagree, somewhat disagree or strongly disagree)

### Parents’ health-promoting behaviour

The majority of parents agree that maternal and paternal health-promoting behaviour is important for a child’s health during preconception (maternal: 89%, paternal: 85%), during pregnancy (maternal: 97%, paternal: 88%) and during the first 2 years of childhood (maternal: 97%, paternal: 96%). Parents more often agree that maternal and paternal health-promoting behaviour during pregnancy and during childhood is important for a child’s general health than during preconception (*P* < .001, *P =* .001, *P <* .001, *P <* .001, respectively) (see Table 7).

Similarly, the majority of professionals agree that maternal and paternal health-promoting behaviour is important for a child’s health during preconception (maternal: 96%, paternal: 91%), during pregnancy (maternal: 98%, paternal: 94%) and during the first 2 years of childhood (maternal: 99%, paternal: 99%). Professionals more often agree that maternal and paternal health-promoting behaviour during pregnancy and during childhood is important for a child’s general health than during preconception (*P =* .022, *P =* .027, *P =* .006, *P =* .018, respectively) (see Table 8).

### The first 1000 days

The term ‘the first 1000 days’ is known by 51% of parents and 70% of professionals. The majority of parents (93%) and professionals (98%) agree that ‘the first 1000 days are important’.

**Table 8:** Differences in proportion of professionals’ agreement regarding the importance of parents’ health-promoting behaviour during preconception, pregnancy and childhood for a child’s (future) health and development.

	Preconception	Pregnancy	Sig.	Preconception	Childhood	Sig.	Pregnancy	Childhood	Sig.
It is important that a mother choses behaviour that promotes a (future) child’s health and development during^a^:	277 (95.5%)	285 (98.3%)	*p* = 0.022	277 (95.5%)	286 (98.6%)	*p* = 0.006	285 (98.3%)	286 (98.6%)	*p* = 0.625
It is important that a father choses behaviour that promotes a (future) child’s health and development during^b^:	262 (90.7%)	272 (94.1%)	*p* = 0.027	262 (90.7%)	285 (98.6%)	*p* = 0.018	272 (94.1%)	285 (98.6%)	*p* < 0.001

a 32 missing values

b 33 missing values

^*^All values are percentages of parents stating strongly agree and somewhat agree (compared with neither agree nor disagree, somewhat disagree or strongly disagree)

**Table 9:** Association of parents’ socio-demographic characteristics and their agreement during preconception regarding the importance of parents’ general health and psychosocial and lifestyle factor for a child’s (future) health outcomes.

	B	S.E.	Exp(B)	95% C.I.		Sig.
Current intention to conceive						0.037
Current pregnancy	2.057	1.072	7.826	0.957	63.998	0.055
No current intention to conceive and no pregnancy	0.814	0.368	2.256	1.096	4.644	0.027
**Constant**	1.526	0.349	4.600			<0.001

a Results of the final model from the multivariate logistic regression using the backward stepwise Wald method.

**Table 10:** Association of parents’ socio-demographic characteristics and their agreement during pregnancy regarding the importance of parents’ general health and psychosocial and lifestyle factors for a child’s (future) health outcomes.

	B	S.E.	Exp(B)	95% C.I.		Sig.
Age (29 years or younger)						0.038
Age (30-34 years)	−0.381	0.193	0.683	0.468	0.996	0.048
Age (35-39 years)	−0.355	0.199	0.701	0.475	1.034	0.073
Age (40 years or older)	−0.774	0.270	0.461	0.272	0.783	0.004
Education partner (post-secondary degree)						0.067
Education partner (undergraduate degree)	−0.205	0.153	0.814	0.603	1.100	0.180
Education partner (university degree)	−0.354	0.153	0.702	0.521	0.947	0.021
**Constant**	0.752	0.173	2.122			<0.001

a Results of the final model from the multivariate logistic regression using the backward stepwise Wald method.

**Table 11:** Association of parents’ socio-demographic characteristics and their agreement during childhood regarding the importance of parents’ general health and psychosocial and lifestyle factors for a child’s (future) health outcomes.

	B	S.E.	Exp(B)	95% C.I.		Sig,
Education (post-secondary degree)						0.011
Education (undergraduate degree)	−0.381	0.214	0.683	0.449	1.040	0.075
Education (university degree)	−0.687	0.233	0.503	0.319	0.793	0.003
Education partner (post-secondary degree)						0.065
Education partner (undergraduate degree)	−0.085	0.186	0.919	0.638	1.324	0.650
Education partner (university degree)	−0.424	0.199	0.654	0.443	0.966	0.033
**Constant**	1.600	0.180	4.952			<0.001

^a^Results of the final model from the multivariate logistic regression using the backward stepwise Wald method.

### Differences in agreement between parents and professionals

Professionals more often than parents agree that maternal health during pregnancy is important for a child’s health during adulthood (*P =* .002), childhood (*P <* .001), and pregnancy (*P* < .001). This is similar for paternal health.

### Socio-demographic characteristics and agreement

Tables 9–11 represent the multivariate analyses of the association between parents’ and professionals’ characteristics and their agreement on the importance of parents’ health and psychosocial and lifestyle factors of parents during preconception, pregnancy, and childhood for a child’s health during its life course. For parents, no current wish to conceive or pregnancy is associated with more agreement regarding the importance of these factors during preconception (OR = 2.256; 95% CI, 1.096, 4.644). Moreover, being aged between 30 and 34 or ≥ 40, or being a partner with an undergraduate or university degree is associated with less agreement regarding the importance of these factors during pregnancy (OR = 0.683; 95% CI, 0.468–0.996, OR = 0.461; 95% CI, 0.272–0.783, OR = 0.814; 95% CI, 0.603–1.100, OR = 0.702; 95% CI, 0.521–0.917). Additionally, being a parent or a partner with a university degree is associated with less agreement regarding these factors during childhood (OR = 0.503; 95% CI, 0.319–0.793; OR = 0.654; 95% CI, 0.443–0.966). For professionals, there is no association between their characteristics and their level of agreement.

## Discussion

### Main findings of this study

Our results show that a large majority of parents and professionals recognize the importance of parents’ general health and multiple psychosocial and lifestyle factors of parents during preconception, pregnancy, and childhood for a child’s health throughout the life course—i.e. immediate health during pregnancy and childhood as well as long-term health in adulthood. Additionally, we found that both parents and professionals agree that maternal and paternal health-promoting behaviour during preconception, pregnancy, and childhood is important for a child’s general health.

### What is already known on this topic

Research evaluating perspectives on the importance of ELC-circumstances on a child’s adult health is very limited. Only one systematic review of 10 studies identified varying perspectives, with differing operational definitions complicating comparisons.^19^

### Parents

Two survey studies—one by Lynch et al. in Australia with 391 participants including 265 parents and one by Bagheri et al. in Iran with 135 parents—reported that 17%–31% of participants heard of ‘the first 1000 days’.^25^^,^^26^ Lynch et al. found that 64% of participants considered maternal health before pregnancy very important for a future baby, while 87% acknowledge its importance during pregnancy, and 44% considered paternal health very important.^25^ Moreover, Lynch et al. reported that 46% of participants somewhat agree that the food a woman eats when she is pregnant affects the health of her baby in adulthood.^25^ Among 2071 first-time mothers surveyed in five European countries, 96% recognized that infant feeding in the first few months affect a child’s health during first years, while 89% acknowledged its effects for subsequent years.^27^ Socio-demographic characteristics are associated with parents’ perspectives as higher age, higher education, higher socio-economic status and lower number of previous children are associated with more knowledge of the impact of ELC on a child’s adult health.^26^^,^^28^

### Professionals

Studies in Japan and New Zealand reported that after four years of professional education, 70% of Japanese and 60% of New Zealand nutrition and nursing students were familiar with ‘the first 1000 days’. Awareness of the impact of maternal health before conception on fetal health during pregnancy was significantly higher than awareness on paternal factors across all undergraduate years.^24^ One qualitative study highlighted the importance of educational opportunities for knowledge about the first 1000 days for professionals.^29^

### What this study adds

To our knowledge, this is the first population-based study conducted within a city-wide cohort of parents with a child up to two years and professionals involved in ELC-care. While our findings show that recognition among parents and professionals regarding the importance of parents’ health and psychosocial and lifestyle factors of parents during the ELC is high and the majority of them agrees that parent’s health-promoting behaviour during the ELC is important, our findings align with previous studies indicating that this recognition does not consistently translate into comprehension or behavioural change.^19^

This gap may be *partly* explained by socioeconomic and systemic barriers that limit the possibility to act on recognized health risks, such as limited financial resources and access to healthcare, or lack of social support. Additionally, psychological mechanisms such as *delay discounting*^30^—in which immediate needs are prioritized over long-term benefits—can further complicate decision-making, particularly among families facing adversity. For these parents, short-term survival may outweigh long-term health planning for their child, even when the importance of early-life health is acknowledged.

Addressing this gap represents a significant opportunity for enhancing prevention strategies through effective knowledge translation. The ELC provides a key window of opportunity to reduce future burden of NCDs, with investment in this period yielding substantial economic and societal benefits.^31^ Effective knowledge translation encompasses several critical components, including translating scientific evidence into clear, actionable guidelines that are both accessible to parents and professionals and aligned with their lived experiences.^19^ Furthermore, involving both parents and professionals in co-creating interventions enhances both the relevance and applicability of prevention strategies, thereby increasing the likelihood of successful implementation and sustained behaviour change.^17^ Finally, fostering trust and collaborative relationships between parents and professionals is essential for creating an environment in which health recommendations are not only accepted but actively adopted.^32^

### Limitations of this study

This study has limitations related to (self)-selection bias and social desirability bias. For parents, recruitment through e-mail and a Dutch-language survey may have resulted in underrepresentation of parents with limited Dutch proficiency and low digital literacy. Additionally, the fact that 42.8% of parents (7727) who clicked on the survey did not submit any answers suggest the possibility of selection bias, potentially influenced by varying degrees of interest in the topic, whereby those who are either less or more attuned to ELC-health or actively seeking child development information may have been more inclined to participate. Moreover, missing data arose from participants either skipping questions and/or leaving the survey incomplete. Specifically, for parents, the number of skipped questions per statement ranged from 8 (0.4%) to 184 (9.9%), while for professionals, it ranged from 1 (0.3%) to 35 (10.9%) per statement. The first statement was answered by 1854 parents and 322 professionals, while the final statement was answered by 1397 (75%) parents and 279 (87%) professionals. These factors potentially introduce selection bias. Furthermore, the underrepresentation of fathers limits generalizability of the findings to paternal perspectives, with the final sample predominantly consisting of mothers. Lastly, given that the survey was conducted within a preventive healthcare setting, parents may have responded in a socially desirable manner, potentially aligning their responses with perceived expectations. For professionals, the lack of formal registration led to recruitment through organizations involved in the research collaborative. This may have resulted in an overrepresentation of professionals who are already aware of the importance of ELC. Lastly, among both parents and professionals, high recognition may be been influenced by availability bias, stemming from local, long-standing efforts to translate Developmental Origins of Health and Disease knowledge into actionable programs and public messaging. In Rotterdam, a sustained collaboration between the Department of Obstetrics and the city council—termed ‘social obstetrics’—has focused on addressing health disparities linked to socio-economic differences across neighbourhoods. This efforts have led to initiatives such as Mothers of Rotterdam, Healthy Pregnancy for All, Connect2Grow, as well as the nationwide Solid Start action program launched by the Dutch Ministry of Health, Welfare and Sport, which integrates medical and social support during the ELC. These initiatives may therefore limit the generalizability of our findings to other contexts.^33^

### Future research

The results of this study point towards future research directions for enhance preventive care during ELC. First, it is important to capture a broader range of parental perspectives, including those of individuals in underprivileged positions as well as fathers. Moreover, future research is needed to explore more in depth the gap between acknowledging the importance of the ELC for long-term health and acting on that with health-promoting behaviour. In this context, examining barriers related to translating this knowledge into action among individuals in underprivileged positions—particularly how systematic barriers and individual resilience may interact—may be an especially interesting direction. Furthermore, exploration of tailored, co-created interventions and approaches that emphasize relational trust and contextual relevance might be particularly promising. Additionally, while this study used a survey-based approach, future research could benefit from qualitative methods, such as ethnographic or participatory designs, to gain a deeper understanding of how families navigate the ELC in their everyday contexts. Such approaches could also inform the development of more grounded, context-sensitive interventions.

## Conclusion

The majority of parents with a child up to two years and maternal and child healthcare professionals recognize the importance of parents’ general health and well-being, parents’ health-promoting behaviour and multiple psychosocial and lifestyle factors of parents during the ELC for a child’s immediate health and well-being during pregnancy and childhood as well as for a child’s long-term health and well-being in adulthood.

## Supplementary Material

Appendix_A_fdaf133

Table_5-16_Black_and_White_Supplement_fdaf133

## Data Availability

The data underlying this article will be shared upon reasonable request by the corresponding author.

## Notes

**Sushma C. Munshi**, MSc

**Loes C.M. Bertens**, PhD

**Anne Marie Weggelaar-Jansen**, PhD

**Hiske E. Ernst-Smelt**, MSc

**Mijke P. Lambregtse-van den Berg**, MD PhD

**Hanneke W. Harmsen van der Vliet-Torij**, PhD

**Eric A.P. Steegers**, MD PhD

**Hilmar H. Bijma**, MD PhD
