# Supplementary material for: Perspectives on the importance of parents’ health, health-promoting behaviour, and psychosocial and lifestyle factors during pregnancy on child health outcomes across the life course: a cross-sectional study among parents and professionals
Source: J Public Health (Oxf). 2025 Oct 22;48(1):109–21. doi: 10.1093/pubmed/fdaf133 (PMC13017653; doi:10.1093/pubmed/fdaf133)
Supplement: Appendix_A_fdaf133 [file appendix_a_fdaf133.docx]

**Appendix A**. Translated survey

*Health and development of a child*

The following questions are about the health and development of a child.

1. **Do you know the term 'the first 1000 days of a child'?**
   Yes
   No
   Skip question
2. **To what extent do you agree with the following statement?**

|  | Strongly agree | Somewhat agree | Neither agree nor disagree | Somewhat disagree | Strongly disagree | Skip question |
| --- | --- | --- | --- | --- | --- | --- |
| The first 1000 days of a child are important |  |  |  |  |  |  |

______________________________________ Next page ________________________________________

1. **To what extent do you agree with the following statements?**

|  | Strongly agree | Somewhat agree | Neither agree nor disagree | Somewhat disagree | Strongly disagree | Skip question |
| --- | --- | --- | --- | --- | --- | --- |
| Before pregnancy  A **woman’s** health and well-being before pregnancy are important for the health and development of the child **during pregnancy** |  |  |  |  |  |  |
| A man’s health and well-being before pregnancy are important for the health and development of the child **during pregnancy** |  |  |  |  |  |  |

______________________________________ Next page ________________________________________

1. **To what extent do you agree with the following statements?**

|  | Strongly agree | Somewhat agree | Neither agree nor disagree | Somewhat disagree | Strongly disagree | Skip question |
| --- | --- | --- | --- | --- | --- | --- |
| During pregnancy  A **woman’s** health and well-being during pregnancy are important for the health and development of the child **during pregnancy** |  |  |  |  |  |  |
| A **man’s** health and well-being during pregnancy are important for the health and development of the child **during pregnancy** |  |  |  |  |  |  |
| A **woman’s** health and well-being during pregnancy are important for the health and development of the child **during childhood** |  |  |  |  |  |  |
| A **man’s** health and well-being during pregnancy are important for the health and development of the child **during childhood** |  |  |  |  |  |  |
| A **woman’s** health and well-being during pregnancy are important for the health and development of the child **in adulthood** |  |  |  |  |  |  |
| A **man’s** health and well-being during pregnancy are important for the health and development of the child **in adulthood** |  |  |  |  |  |  |

______________________________________ Next page ________________________________________

1. **To what extent do you agree with the following statements about the living circumstances (such as poverty, nutrition, chronic stress, neighborhood, parenting) of a child aged 0–2 years?**

|  | Strongly agree | Somewhat agree | Neither agree nor disagree | Somewhat disagree | Strongly disagree | Skip question |
| --- | --- | --- | --- | --- | --- | --- |
| The living circumstances of a child aged 0–2 years are important for the child’s health and development **throughout childhood** |  |  |  |  |  |  |
| The living circumstances of a child aged 0–2 years are important for the child’s health and development **in adulthood** |  |  |  |  |  |  |

______________________________________ Next page ________________________________________
 *Choices of (future) parents*

The following questions are about your opinion on the choices that (future) parents make regarding the health and development of a child.

1. **To what extent do you agree with the following statements?**

|  | Strongly agree | Somewhat agree | Neither agree nor disagree | Somewhat disagree | Strongly disagree | Skip question |
| --- | --- | --- | --- | --- | --- | --- |
| It is important that a **woman** makes choices before pregnancy that support the health and development of her (future) child |  |  |  |  |  |  |
| It is important that a **man** makes choices before pregnancy that support the health and development of his (future) child |  |  |  |  |  |  |
| It is important that a **woman** makes choices during pregnancy that support the health and development of her (future) child |  |  |  |  |  |  |
| It is important that a **man** makes choices during pregnancy that support the health and development of his (future) child |  |  |  |  |  |  |
| It is important that a **woman** makes choices during this first two years of a child’s life that support the health and development of her (future) child |  |  |  |  |  |  |
| It is important that a **man** makes choices during this first two years of a child’s life that support the health and development of his (future) child |  |  |  |  |  |  |

**Below you can explain your answer to one of the above statements. You can also share your opinion about the choices (future) parents make regarding the health and development of a child.**

______________________________________ Next page ________________________________________

1. **Below are circumstances before pregnancy. To what extent do you agree with the following statements?**

|  | Strongly agree | Somewhat agree | Neither agree nor disagree | Somewhat disagree | Strongly disagree | Skip question |
| --- | --- | --- | --- | --- | --- | --- |
| Folic acid use by **the (future) mother** before pregnancy influences the health and well-being of the child in adulthood |  |  |  |  |  |  |
| Folic acid use by **the (future) father** before pregnancy influences the health and well-being of the child in adulthood |  |  |  |  |  |  |
| Nutrition of **the (future) mother** before pregnancy influences the health and well-being of the child in adulthood |  |  |  |  |  |  |
| Nutrition of **the (future) father** before pregnancy influences the health and well-being of the child in adulthood |  |  |  |  |  |  |

_____________________________________ Next page _________________________________________

1. **To what extent do you agree with the following statement?**

**Statement:** **The following living circumstances of the pregnant individual during pregnancy influence the health and well-being of the child in adulthood.**

|  | Strongly agree | Somewhat agree | Neither agree nor disagree | Somewhat disagree | Strongly disagree | Skip question |
| --- | --- | --- | --- | --- | --- | --- |
| Healthy nutrition |  |  |  |  |  |  |
| Smoking |  |  |  |  |  |  |
| Cannabis/hard drug use |  |  |  |  |  |  |
| Alcohol use |  |  |  |  |  |  |
| Overweight |  |  |  |  |  |  |
| Severe and persistent stress |  |  |  |  |  |  |
| Poverty |  |  |  |  |  |  |
| Living in an underprivileged neighborhood |  |  |  |  |  |  |

_____________________________________ Next page _________________________________________

1. **To what extent do you agree with the following statement?**

**Statement:** **The following living circumstances of a child aged 0–2 years influence the health and well-being of the child in adulthood.**

|  | Strongly agree | Somewhat agree | Neither agree nor disagree | Somewhat disagree | Strongly disagree | Skip question |
| --- | --- | --- | --- | --- | --- | --- |
| Being born prematurely |  |  |  |  |  |  |
| Being small at birth |  |  |  |  |  |  |
| Being large at birth |  |  |  |  |  |  |
| Breastfeeding |  |  |  |  |  |  |
| Smoking in the home |  |  |  |  |  |  |
| Severe and persistent parental stress |  |  |  |  |  |  |
| A good parent–child relationship |  |  |  |  |  |  |
| Psychological, emotional, or physical abuse of the child |  |  |  |  |  |  |
| Psychological, emotional, or physical neglect of the child |  |  |  |  |  |  |
| Overweight child |  |  |  |  |  |  |
| Poverty |  |  |  |  |  |  |
| Living in an underprivileged neighborhood |  |  |  |  |  |  |
